# INVESTIGATING WITHIN-PERSON MEDIATION OF GAIT-COGNITION ASSOCIATIONS IN A WALKING INTERVENTION FOR OLDER ADULTS

**DOI:** 10.1093/geroni/igae098.3192

**Published:** 2024-12-31

**Authors:** Nicholas Tamburri, Cynthia McDowell, Sandra Hundza, Stuart MacDonald

**Affiliations:** University of Victoria, Victoria, British Columbia, Canada; University of Victoria, Victoria, British Columbia, Canada; University of Victoria, Victoria, British Columbia, Canada; University of Victoria, Victoria, British Columbia, Canada

## Abstract

**Objective:**

Between-person associations between gait and cognitive function are well-documented, with select interventions (e.g., exercise) known to benefit both physiological function and cognitive health. Comparatively few studies, however, have investigated links between within-person changes in gait and cognition in the context of exercise interventions, or the mechanisms that might underlie this relationship. Method: The current study employed data from the Healthy Bodies Healthy Minds study -- a longitudinal walking intervention for initially-sedentary older adults (N = 118, 65-87 years). The study employs an intensive repeated-measures design, with physiological and cognitive function indexed at 5 assessments: baseline, 6, 9, 12, and 16 weeks. Gait velocity was assessed using a GAITRite Computerized Walkway, executive functioning was measured via the Groton Maze Learning (GML) Test, with aerobic capacity indexed by converting performance on the Rockport 1-Mile Walk Test to a V02 max proxy.

**Results:**

Employing longitudinal multilevel mediation (1-1-1) models, we explored the within-person relationship between gait velocity and executive functioning, with particular consideration of aerobic capacity as a mediator. The indirect effect (ab+σajbj=-0.25) was significant, with aerobic capacity mediating 14.5% of the within-person gait-cognition time-varying association. Consistent with hypotheses, increases in gait velocity were associated with increases in aerobic capacity, which, in turn, conferred downstream benefits on GML test performance. Conclusions: These results further our understanding of the gait-cognition relationship, identifying aerobic health as an important within-person mediator in the context of a walking intervention. Key implications include the potential benefits of simple lifestyle interventions for promoting cognitive health in older adults.

